# The Relationship between Tumor Development and Sarcoidosis in Aspects of Carcinogenesis before and after the Onset of Sarcoidosis

**DOI:** 10.3390/medicina58060768

**Published:** 2022-06-06

**Authors:** Yoshimasa Hachisu, Yasuhiko Koga, Shu Kasama, Kyoichi Kaira, Shogo Uno, Masakiyo Yatomi, Haruka Aoki-Saito, Hiroaki Tsurumaki, Asuka Jingu, Noriaki Sunaga, Toshitaka Maeno, Takeshi Hisada

**Affiliations:** 1 Department of Respiratory Medicine, Gunma University Graduate School of Medicine, Maebashi 371-8511, Japan; yhachisu2002@yahoo.co.jp (Y.H.); s.uno0120@icloud.com (S.U.); m09702007@gunma-u.ac.jp (M.Y.); a-haruka@gunma-u.ac.jp (H.A.-S.); m12702056@gunma-u.ac.jp (H.T.); nsunaga@gunma-u.ac.jp (N.S.); mutoyu03@gunma-u.ac.jp (T.M.); 2 Department of Respiratory Medicine, Maebashi Red Cross Hospital, Maebashi 371-0813, Japan; tamaki.ja.schwarz@gmail.com; 3 Institute for Clinical and Translational Science, Nara Medical University Hospital, Nara 634-8522, Japan; s-kasama@bay.wind.ne.jp; 4 Department of Respiratory Medicine, Comprehensive Cancer Center, International Medical Center, Saitama Medical University, Saitama 350-1298, Japan; kkaira1970@yahoo.co.jp; 5 Gunma University Graduate School of Health Sciences, Maebashi 371-8514, Japan; hisadat@gunma-u.ac.jp

**Keywords:** sarcoidosis, Th17, cancer, immune checkpoint inhibitor, Th1

## Abstract

*Background and Objectives*: It is still unclear whether sarcoidosis is likely to be associated with tumors. In addition, the use of an immune checkpoint inhibitor has been reported to initiate the onset of sarcoidosis. We retrospectively analyzed tumor development before and after the diagnosis of sarcoidosis and examined the impact of having a history of tumors on the activity or the severity of sarcoidosis. *Materials and Methods*: We recruited 312 consecutive cases of sarcoidosis and analyzed the tumor development before and after the onset of sarcoidosis. *Results*: Among them, 25 cases were diagnosed with malignant tumor after diagnosis of sarcoidosis. In the analysis of the tumor-development group after diagnosis of sarcoidosis, both serum angiotensin I-converting enzyme and mediastinal lymph node size were significantly reduced at the time of malignant tumor diagnosis compared to at the onset of sarcoidosis, indicating that the decreasing activity of sarcoidosis may be partly associated with tumor development. Furthermore, we examined 34 cases having tumor history before the onset of sarcoidosis and analyzed the effect of tumor history on the severity of sarcoidosis. Cases with a malignant tumor in the past were older and had less complicated organs of sarcoidosis than cases without malignant tumors in the past. Oral corticosteroid therapy was administrated more frequently in cases without malignant tumors in the past, indicating that the history of a malignant tumor may influence the severity of sarcoidosis. *Conclusion*: These results indicate that tumor development may be partly associated with the activity or severity of sarcoidosis.

## 1. Introduction

Sarcoidosis is an unexplained systemic granulomatous disease. Occupational exposure to silica dust increases the risk of sarcoidosis in younger men [1]. The risks for sarcoidosis have been suggested to correlate with occupational inorganic dust exposures, such as silica/silicates, and metals [2,3]. We detected inorganic dust in the lungs and hilar lymph nodes [4,5] and suggested the relationship between the progression of idiopathic pulmonary fibrosis and silica/silicates accumulation in the lungs by in-air micro particle-induced X-ray emission analysis [6]. It remains unclear as to whether there is any relationship between sarcoidosis and malignant tumors. Furthermore, there are few reports regarding the effect of tumor immunity on sarcoidosis. Recently, an immune checkpoint inhibitor (ICI) was reported to initiate the onset of sarcoidosis [7,8,9,10,11,12,13,14]. Sarcoidosis is an idiopathic granulomatous disease and develops across age groups. It is known that the T helper (Th)1-dominant immune reaction occurs due to the involvement of acne bacteria, acid-fast bacteria with granulomatous formation [15,16,17]. Granulomas occur in various organs such as the eyes, lungs, heart, skin, and nerves, causing various symptoms. There have been several reports on the relationship between sarcoidosis and tumors in regard with Th1/Th2/Th17 immune balance, but a significant relationship has not yet been concluded [18,19,20,21].

The advent of ICI treatment is advancing research into the development of Th17 balance-mediated sarcoidosis. Furthermore, epidemiologically, research on silica exposure and the development of sarcoidosis is also progressing [2]. Not only idiopathic pulmonary fibrosis [6,22] but also ANCA-related vasculitis associated with interstitial pneumonia and systemic scleroderma have long been reported to be associated with silica exposure [23,24,25,26,27]. Studies that consider multiple factors, including tumor immunity and environmental exposure, may be useful in elucidating the etiology of sarcoidosis.

Investigations regarding sarcoidosis along with a history of malignant tumors have been limited. In this study, we retrospectively analyzed tumor development before and after the diagnosis of sarcoidosis and examined the impact of having a history of tumors on the activity or the severity of sarcoidosis.

## 2. Materials and Methods

### 2.1. Study Population

Consecutive patients diagnosed with sarcoidosis at Gunma University Hospital were recruited retrospectively. We applied the opt-out method to obtain consent for this study by declaring an information disclosure statement. The information disclosure statement was approved by the Gunma University Institutional Review Board. Patients were diagnosed with sarcoidosis by multiple respiratory specialists in this study.

A total of 312 consecutive patients were enrolled in this study from those that were diagnosed with sarcoidosis at our hospital between January 2007 to December 2017. We excluded two cases in which malignant tumors and sarcoidosis were diagnosed at the same time to exclude sarcoid reactions. The diagnostic criteria of sarcoidosis were based on the statement of American Thoracic Society (ATS)/European Respiratory Society (ERS)/World Association of Sarcoidosis and Other Granulomatous Disorders (WASOG) [28,29]. Broncho-alveolar lavage fluid (BALF) and trans-bronchial lung biopsy were performed for the initial diagnosis of sarcoidosis. We examined serum neuron-specific enolase, progastrin-releasing peptide, and Krebs von den Lungen-6 biomarkers to evaluate the possibility of small cell lung cancer and interstitial lung disease. This study was conducted with the approval of the Ethics Review Committee of Gunma University Hospital, No. 2017-048.

### 2.2. Clinical Assessment

Statistical information was collected from the medical record, including patient’s age, sex, height, weight at the time of diagnosis, hematological data on the course of treatment, imaging, allergy history, and preference history, which may potentially affect the course of sarcoidosis. Patient’s body mass index (BMI) was calculated using their height and weight at the time of analysis. Alcohol history defined patients’ daily consumption habits. A history of smoking was defined as an individual who currently smokes or has smoked previously more than 100 cigarettes in total [30]. Methotrexate, azathioprine, and tacrolimus were used as immunosuppressants. The size of lymph nodes was evaluated by one respiratory specialist who has an experience as a pulmonologist for more than 5 years.

Most patients underwent contrast-free computed tomography (CT). It was difficult to measure hilar lymph nodes because the borderline between hilar lymph nodes and blood vessels was unclear. Therefore, we evaluated the most measurable lymph nodes, # 4 and 7, as lesions there can be measured by contrast-free CT. The measurements of lymph nodes were performed according to resist criteria 1.1 [31].

### 2.3. Statistical Analysis

Cases with tumor development/history of a tumor (tumor group) and without tumor development/history of a tumor (non-tumor group) were divided into groups. For each factor of the tumor group and the non-tumor group, the number of cases and the ratio were calculated on the nominal and average scale, and the standard deviation was calculated on the order scale. Missing data were replaced by dummy variables. The Fisher’s accuracy test was used to analyze the nominal scale, while the numerical scale used the Mann–Whitney’s test. A *p*-value of <0.05 was considered statistically significant. All analyzes were done using SPSS version 24 (IBM, Armonk, NY, USA).

## 3. Results

### 3.1. Analysis of Cases with Malignancy after Diagnosis of Sarcoidosis

First, we analyzed the relationship between the activity of sarcoidosis and the development of malignant tumors. Of the 312 sarcoidosis patients, 25 cases were later diagnosed with malignancies. In our study, lung cancer was the most common in seven cases, followed by malignant lymphoma in four cases (Table 1). One case had two types of cancer after diagnosis of sarcoidosis.

A statistical comparison of the background of patients did not show any significant differences between the tumor development group and the non-tumor development group (Table 2).

### 3.2. Relationship between the Activity of Sarcoidosis and the Development of Malignant Tumors

We analyzed the relationship between the activity of sarcoidosis and the development of malignant tumors. Serological data in patients with sarcoidosis having tumor development showed that serum angiotensin I-converting enzyme (ACE) was significantly reduced at the onset of malignant tumors compared to at the time of diagnosis of sarcoidosis. The major and minor diameters of the mediastinal lymph nodes were measured. The major and minor diameters of lymph node # 7 and the minor diameters of lymph node # 4 were significantly reduced at the diagnosis of malignant tumors compared to the size of initial lymphadenopathy at the onset of sarcoidosis (Table 3). Lymph node swelling did not affect the diagnosis of cancer staging in all seven patients who developed lung cancer after the diagnosis of sarcoidosis. Comparison with past CT images distinguished between lymphadenopathy due to sarcoidosis and lymphadenopathy due to lung cancer. The preoperative diagnosis was consistent with the postoperative diagnosis.

### 3.3. Analysis of Cases with Tumor History before Diagnosis of Sarcoidosis

Next, we analyzed the effect of tumor history on the development of sarcoidosis. Thirty-four cases had a history of having a malignant tumor in the past (past-tumor group; 10.9%). One case had a history of two types of cancer in the past. Tumor types are shown in Table 4. Seven cases had a history of colon cancer, five had uterine cancer, four had breast cancer, and four had prostate cancer.

A statistical comparison of the background of patients was made between the past-tumor group and the non-past-tumor group (Table 5). The age at which a sarcoidosis diagnosis was made was significantly older in the past-tumor groups (60.41 ± 12.80 vs. 53.16 ± 15.83, *p* = 0.009). Diabetes mellitus, smoking history, and alcohol intake were not significantly different between both groups.

Regarding the serological marker when diagnosed with sarcoidosis, serum ACE and soluble Interleukin (IL)-2 receptor tended to be lower in past tumor groups (18.3 ± 5.9 vs. 21.8 ± 13.5, *p* = 0.061, 782 ± 351 vs. 1093 ± 889, *p* = 0.072, respectively). Although we recently reported that serum neuron-specific enolase levels could be a diagnosing and monitoring marker of sarcoidosis, there was no significant difference in serum neuron-specific enolase levels [32]. BALF in the CD4/8 ratio of the past tumor group was significantly higher (8.17 + 5.19 vs. 6.08 + 4.22, *p* = 0.038) (Table 6).

### 3.4. Association of Tumor History with the Severity of Sarcoidosis

Furthermore, we analyzed the severity of sarcoidosis with or without tumor history. A comparison of the affected organs and the severity of sarcoidosis between the past tumor and the non-past-tumor group are shown in Table 7. The past-tumor group had less complicated organs of sarcoidosis, while cases in the non-past-tumor group had frequently had more than three complicated organs (8.8% (*n* = 3) vs. 28.1% (*n* = 78), *p* = 0.013). Furthermore, oral corticosteroid therapy was administrated more frequently in the non-tumor group than in those cases that had a malignant tumor in the past (5.9% (*n* = 2) vs. 23.0% (*n* = 64), *p* = 0.024). There was no significant difference in the follow-up period and mortality.

## 4. Discussion

Sarcoidosis is an unexplained systemic granulomatous disease. Sarcoidosis is characterized by the activation of the Th1 immune response induced by antigen exposure such as *P. acnes* or mycobacteria. Various symptoms are observed in systemic organs such as the eyes, lungs, heart, nerves, and skin, with oral corticosteroids being administrated as needed [17,21]. In this study, compared to the onset of sarcoidosis, decreased serum ACE levels and mediastinal lymph node size at the time of subsequent definitive diagnosis of malignant tumors were observed, indicating that the activity of sarcoidosis may be partially associated with the subsequent carcinogenesis. Recently, we suggested a hypothesis that a remission of sarcoidosis may be linked with carcinogenesis based on the cases of small cell lung cancer complicated with sarcoidosis in the spontaneous remission stage [33]. Consistently, Steinfort et al. also reported that the sarcoid reactions within regional lymph nodes of lung cancer are associated with a lower recurrence rate after surgical resection [34]. Although the relationship between sarcoidosis and cancer remains unclear, the activity of sarcoidosis may be partly linked on carcinogenesis in some cases.

In this study, both the number of patients with systemic oral corticosteroids (OCSs) and the number of affected organs were significantly lower in the past-tumor group. The serum ACE and soluble IL-2 receptor, which reflects the activity of sarcoidosis, showed a decreasing trend in the past-tumor group. To our knowledge, this is first report describing the influence of malignant tumor history on the severity of sarcoidosis. As the number of diseased organs and frequency of OCSs treatment may reflect on the activity of sarcoidosis, immune status after tumor development is possibly associated with the severity of sarcoidosis after its onset.

**Figure 1:**
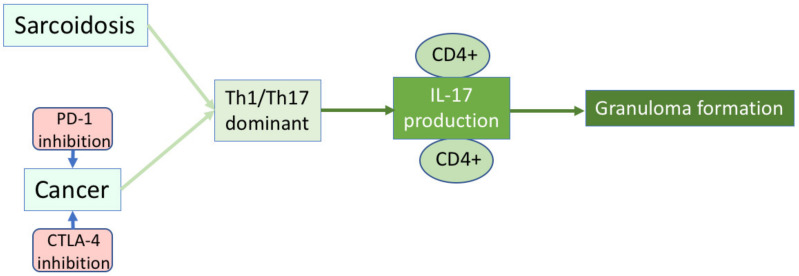
Mechanisms of development of sarcoidosis and ICI-induced sarcoid-like granuloma formation.

There have been several reports on the relationship between sarcoidosis and tumors. Bonifaze et al. reported that skin tumors, blood tumors, upper gastrointestinal tumors, kidney tumors, liver tumors, and colon tumors are more frequently found with sarcoidosis [21]. Askling et al. also reported an increased incidence of non-Hodgkin’s lymphoma, malignant melanoma, and liver cancer with sarcoidosis [19]. However, Rømer et al. found no significant difference in the association between sarcoidosis and tumor development because sarcoidosis may involve a sarcoid reaction [20]. The sarcoid reaction is thought to be introduced by any antigen exposure involved with granuloma formation without any systemic symptoms of sarcoidosis [35]. It has been recently asserted that ICI treatment for cancer, which activates tumor immunity, can induce the onset of sarcoidosis or sarcoid-like granulomas (SLGs) [7,8,9,10,11,12,13,14]. The SLGs can be initiated with the predominance of the Th1 balance by ICI treatment [12]. CTLA4 blockade increases lymphocytes expressing Th1-related markers, thus promoting SLGs [36]. Further, CTLA4 and/or PD-1 blockade may increase the ratio of Th17/T regulatory cells and induce the secretion of IL-17 from CD4^+^ cells [36,37,38] (Figure 1). The role of CD4+ T cells in anti-tumor immunity has been studied in cancer patients. CD4+ T cells are critical for priming of tumor-specific CD8^+^ T cells [39,40]. In this study, the CD4/8 ratio in the BALF was significantly higher in the past-tumor group than in the non-past-tumor group in patients with sarcoidosis. Sarcoidosis with a history of tumors may have a more similar Th17 immune balance as ICI treatment compared to the group without tumor history.

Th17-dominant CD4-positive T cells are abundant in BALF and granulomatous lesions of sarcoidosis [41,42]. Th17 is also dominant when tumor immunity is activated during immune checkpoint inhibitor treatment. It is speculated that a state of active sarcoidosis with a high Th17 balance is a similar immune state in which tumor immunity is activated under ICI treatment. In this study, sarcoidosis was relieved, and the Th17 balance was lowered; then, malignant tumors tended to develop. The results that malignant tumors were more likely to occur in a remission stage of sarcoidosis suggests that tumor immunity mediated by Th17 balance may be involved in the development of sarcoidosis.

Interestingly, a transition from Th1- to Th2-dominant balance is thought to be particularly relevant in the development of advanced pulmonary sarcoidosis [43]. The shift of the Th1/Th2 balance may affect the severity of sarcoidosis in patients with a history of the tumors. Kokturk et al. reported that sarcoidosis might be associated with a lower incidence of Th2 disorders, such as atopy and allergic disease [44]. Beutler and Cohen suggested pathogenesis of cancer, such as autoimmune mechanism, ultraviolet radiation mechanism, immunologic mechanism, inflammation/antigen response mechanism, or antineoplastic therapy mechanism, in sarcoidosis patients [45].

There are several limitations to this study. Firstly, this was a retrospective study at a single facility, and as such, the number and the background of the cases were limited. A multicenter prospective study with a greater number of sarcoidosis cases will be required to clarify the relationship between sarcoidosis and tumor immunity. Furthermore, this was a cohort study of patients with sarcoidosis but not with tumors. Future analysis on a cohort of patients with tumors is also required to elucidate the relationship between the severity of sarcoidosis and tumor history.

## 5. Conclusions

Serum ACE levels and mediastinal lymph node size were significantly reduced at subsequent tumor occurrence. Past-tumor history in patients with sarcoidosis was associated with a lower rate of OCS administration and fewer organ complications. Tumor development may be partly associated with the pathology or severity of sarcoidosis with regard to anti-tumor immunity.

## Figures and Tables

**Table 1:** Types of tumor after diagnosis of sarcoidosis.

Types of Tumor after Diagnosis of Sarcoidosis	Number (*n*)	Period (Year) from the Onset of Sarcoidosis to the Tumor Development	Treatment for Tumor
Lung cancer	7	1.5, 4, 5, 7, 8, 10, 16	Surgery (*n* = 5), chemotherapy (*n* = 1), radiation (*n* = 1)
Malignant lymphoma	4	6, 7, 10, 14	Chemotherapy (*n* = 3), radiation (*n* = 1)
Liver cancer	3	3, 12, 14	Surgery+ TACE (*n* = 2), RFA+ TACE (*n* = 1)
Gastric cancer	2	3, 44	Endoscopic resection *(n* = 1), surgery (*n* = 1)
Brain cancer	2	2, 26	Surgery
Thyroid cancer	1	1	Surgery
Esophageal cancer	1	3	Radiation
Colon cancer	1	38	Surgery
Gum cancer	1	1	Surgery
Breast cancer	1	17	Surgery + chemotherapy
Uterine cancer	1	6	Surgery
Soft tissue tumor	1	2	Surgery
Cancer of unknown primary	1	10	Chemotherapy

**Table 2:** A statistical comparison of the background of patients between the tumor development group and the non-tumor development group.

Background	Tumor Development (*n* = 25)	Non-Tumor Development (*n* = 287)	*p*-Value	Odds Ratio
Age diagnosed sarcoidosis	56.16 ± 16.86	53.76 ± 15.99	0.462	1.01 (0.98–1.04)
Male	24.00%	32.40%	0.389	0.66 (0.26–1.70)
BMI	23.90 ± 4.17	22.47 ± 3.74	0.092	1.09 (0.99–1.21)
DM	4.0% (*n* = 1)	4.5%(*n* = 13)	0.902	0.88 (0.11–7.00)
Dialysis	4.0% (*n* = 1)	0.7% (*n* = 2)	0.152	5.94 (0.52–67.87)
Asthma	8.0% (*n* = 2)	5.9% (*n* = 17)	0.678	1.38 (0.30–6.35)
Allergy to pollen	20.0% (*n* = 5)	10.1% (*n* = 29)	0.161	2.13 (0.74–6.14)
Allergy	24.0% *(n* = 6)	19.9% (*n* = 57)	0.699	1.21 (0.46–3.19)
food allergy	8.0% (*n* = 2)	5.2% (*n* = 15)	0.6	1.51 (0.32–7.03)
drug allergy	20.0% (*n* = 5)	14.6% (*n* = 42)	0.534	1.39 (0.49–3.93)
Smoking	32.0% (*n* = 8)	34.1% (*n* = 98)	0.887	0.94 (0.37–2.34)
Alcohol	16.0% (*n* = 4)	32.8% (*n* = 94)	0.187	0.46 (0.14–1.46)
Foreign	0.0% (*n* = 0)	0.3% (*n* = 1)	1	-
Use of immunosuppressive drugs	0.0% (*n* = 0)	1.4% (*n* = 4)	1	-

Values are mean ± standard deviation (SD) or percentage (%) and number or odds ratio and 95% confidence interval. BMI, body mass index; DM, diabetes mellitus. Immunosuppressive drugs: azathioprine, methotrexate, and tacrolimus.

**Table 3:** Comparison of serological data and mediastinal lymph node size in patients with tumor development following sarcoidosis at the time between the onset of sarcoidosis and the subsequent diagnosis of malignancy.

Serological marker	When diagnosed as sarcoidosis	When found cancer	*p*-value
Alb (*n* = 17) (g/dL)	4.22 ± 0.46	3.91 ± 0.73	0.127
LDH (*n* = 19) (U/L)	191 ± 27	221 ± 112	0.26
ACE (*n* = 16) (IU/L)	21.4 ± 10.7	14.9 ± 6.7	0.007 **
sIL2-R (*n* = 5) (U/mL)	1128 ± 921	560 ± 223	0.181
Ca (*n* = 17) (mg/dL)	9.59 ± 0.48	9.75 ± 0.44	0.436
KL-6 (*n* = 5) (U/mL)	2168 ± 2185	1403 ± 1775	0.06
NSE (*n* = 3) (ng/mL)	13.0 ± 4.4	12.7 ± 4.2	0.827
ProGRP (*n* = 3) (pg/mL)	37.4 ± 14.4	43.9 ± 8.5	0.521
Diameter of lymph node	When diagnosed as sarcoidosis (mm)	When found cancer (mm)	*p*-value
#4R major diameter (*n* = 12)	22.58 ± 6.60	17.47 ± 4.86	0.053
#4R minor diameter (*n* = 12)	14.19 ± 4.46	10.03 ± 3.05	0.005 **
#7 major diameter (*n* = 12)	29.64 ± 12.52	20.46 ± 7.86	0.023 *
#7 minor diameter (*n* = 12)	13.83 ± 6.56	8.59 ± 4.29	0.006 **

LDH, lactate dehydrogenase; ACE, angiotensin I-converting enzyme; sIL-2R, soluble interleukin-2 receptor; KL-6, Krebs von den Lungen-6; NSE, neuron-specific enolase; ProGRP, progastrin-releasing peptide; *p* < 0.05 *, *p* < 0.01 **. Values are mean ± standard deviation.

**Table 4:** Types of past tumors in patients with sarcoidosis.

Type of Past Tumor	Number	Years before Sarcoidosis Diagnosis	Tumor Treatment
Colon cancer	7	4 (0.25–10)	Surgery 6, endoscopic resection 1
Uterine cancer	5	3 (0.75–29)	Surgery 1, surgery + radiation 1, surgery + chemotherapy 3
Breast cancer	4	4 (0.75–9)	Surgery 2, surgery + chemotherapy 2
Prostate cancer	4	4.5 (1–6)	Surgery 1, surgery + chemotherapy 1, chemotherapy + radiation 1, chemotherapy 1
Thyroid cancer	3	0.5 (0.08–1)	Surgery 3
Ovarian cancer	2	2.88 (0.75–5)	Surgery 1, surgery + chemotherapy 1
Malignant melanoma	2	0.33 (0.17–0.5)	Surgery 1, surgery + chemotherapy 1
Stomach cancer	2	8.5 (1–16)	Surgery 2
Adrenal tumor	1	0.08	Surgery 1
Vestibular tumor	1	0.08	No treatment
Follicular dendritic cell sarcoma	1	5	Surgery 1 (Left lower lobectomy)
Nasopharyngeal cancer	1	0.5	Chemo-radiotherapy 1
Oropharyngeal cancer	1	0.17	Chemo-radiotherapy 1
Renal cell carcinoma	1	10	Surgery 1

Values are median and minimum and maximum values.

**Table 5:** Comparison between patients with sarcoidosis that did or did not have a tumor history.

Background	Past Tumor (*n* = 34)	Not Past Tumor (*n* = 278)	*p*-Value	Odds Ratio
Age diagnosed sarcoidosis	60.41 ± 12.80	53.16 ± 15.83	0.009 **	1.04 (1.01–1.06)
Male	29.4% (10)	32.0% (89)	0.847	0.89 (0.41–1.93)
BMI	23.17 ± 2.96	22.54 ± 3.91	0.259	1.04 (0.94–1.15)
DM	11.8% (4)	3.6% (10)	0.054	3.57 (1.06–12.10)
Dialysis	0.0% (0)	1.1% (3)	1	-
Asthma	0.0% (0)	6.8% (19)	0.243	-
Allergy to pollen	5.9% (2)	11.5% (32)	0.557	0.45 (0.10–1.97)
Allergy	23.5% (8)	19.8% (55)	0.651	1.16 (0.50–2.72)
food allergy	2.9% (1)	5.8% (16)	0.705	0.47 (0.06–3.64)
drug allergy	20.6% (7)	14.4% (40)	0.318	1.45 (0.59–3.56)
Smoking	38.2% (13)	33.5% (93)	0.571	1.12 (0.52–2.39)
Alcohol	23.5% (8)	32.4% (90)	0.334	0.48 (0.21–1.13)
Foreign	0.0% (0)	0.4% (1)	1	-
Use of immunosuppressive drugs	2.9% (1)	1.1% (3)	0.371	2.78 (0.28–27.48)

BMI, body mass index; DM, diabetes mellitus. Values are mean ± standard deviation (SD) or percentage (%) and number or odds ratio and 95% confidence interval. ** *p* < 0.01. Immunosuppressive drugs: azathioprine, methotrexate, and tacrolimus.

**Table 6:** Laboratory findings of those with or without a tumor history in patients with sarcoidosis.

Laboratory Data	Past Tumor (*n* = 34)	Not Past Tumor (*n* = 278)	*p*-Value	Odds Ratio
Albumin (g/dL)	4.13 ± 0.36	4.21 ± 0.40	0.303	0.56 (0.22–1.44)
LDH (U/L)	192 ± 39	191 ± 40	0.944	1.00 (0.99–1.01)
ACE (IU/L)	18.3 ± 5.9	21.8 ± 13.5	0.061	0.96 (0.91–1.00)
Lysozyme (μg/mL)	10.3 ± 3.2	12.5 ± 6.9	0.162	0.92 (0.84–1.02)
Soluble IL-2 receptor (U/mL)	782 ± 351	1093 ± 889	0.072	1.00 (1.00–1.00)
Calcium (mg/dL)	9.62 ± 0.43	9.65 ± 0.45	0.646	0.86 (0.38–1.94)
KL-6 (U/mL)	436 ± 193	584 ± 857	0.795	1.00 (1.00–1.00)
NSE (ng/mL)	11.8 ± 5.8	12.3 ± 7.3	0.772	0.99 (0.90–1.09)
ProGRP (pg/mL)	50.0 ± 32.1	42.6 ± 14.1	0.749	1.02 (0.99–1.05)
BALF CD4/8 ratio	8.17 ± 5.19	6.08 ± 4.22	0.038 *	1.09 (0.99–1.20)

LDH, lactate dehydrogenase; ACE, angiotensin-converting enzyme; IL-2, Interleukin-2, KL-6: Krebs von den Lungen-6; NSE, neuron-specific enolase; ProGRP, pro-gastrin-releasing peptide; BALF, bronchoalveolar lavage fluid. Values are mean ± standard deviation (SD) or odds ratio and 95% confidence interval. * *p* < 0.05.

**Table 7:** Number of affected organs (>3) and frequency of systemic therapy with or without tumor history.

Diagnosis	Past Tumor (*n* = 34)	Not Past Tumor (*n* = 278)	*p*-Value	OR
Tissue diagnosis	64.7% (22)	61.5% (171)	0.852	1.14 (0.54–2.39)
Over 3 organ	8.8% (3)	28.1% (78)	0.013 *	0.25 (0.07–0.83)
Systemic therapy	5.9% (2)	23.0% (64)	0.024 *	0.21 (0.05–0.89)
**Progress**	**Past Tumor (*n* = 34)**	**Not Past Tumor (*n* = 278)**	***p*-Value**	**OR**
Follow up time (month)	41.0 ± 35.3	47.5 ± 45.1	0.631	
Tumor after diagnosed with sarcoidosis	8.8% (3)	7.9% (22)	0.744	1.13 (0.32–3.98)
Death	5.9% (2)	1.4% (4)	0.131	4.28 (0.75–24.31)

Values are mean ± standard deviation (SD) or odds ratio and 95% confidence interval. * *p* < 0.05.

## Data Availability

The data presented in this study are available on request from the corresponding author. The data are not publicly available due to privacy.
